# *Trichomonas vaginalis* infection is uncommon in the British general population: implications for clinical testing and public health screening

**DOI:** 10.1136/sextrans-2016-052660

**Published:** 2016-09-29

**Authors:** Nigel Field, Soazig Clifton, Sarah Alexander, Catherine A Ison, Rumena Khanom, Pamela Saunders, Gwenda Hughes, Laura Heath, Simon Beddows, Catherine H Mercer, Clare Tanton, Anne M Johnson, Pam Sonnenberg

**Affiliations:** 1 Research Department of Infection and Population Health, University College London, London, UK; 2 Public Health England, National Centre for Infectious Disease Surveillance and Control (CIDSC), London, UK; 3 Sexually Transmitted Bacteria Reference Unit, Microbiological Services, Public Health England, London, UK; 4 Virus Reference Department, Public Health England, London, UK

**Keywords:** TRICHOMONAS, SCREENING, DIAGNOSIS, PUBLIC HEALTH, INFECTION CONTROL

## Abstract

**Introduction:**

Variable use of new molecular assays, asymptomatic infections and a lack of population data mean that the population burden of *Trichomonas vaginalis* is uncertain. We investigated the age-specific prevalence of *T. vaginalis* within the sexually active British general population to inform testing strategies.

**Methods:**

Britain's third National Survey of Sexual Attitudes and Lifestyle (Natsal-3) is a probability sample survey of 15 162 individuals aged 16–74 years, undertaken during 2010–2012. Urine from 4386 participants aged 16–44 years reporting ≥1 lifetime sexual partner was tested for *T. vaginalis* using in-house real-time PCR.

**Results:**

Urinary *T. vaginalis* was detected in seven women and no men providing urine samples, giving a weighted prevalence estimate of 0.3% (95% CI 0.1% to 0.5%) in sexually experienced women aged 16–44 years. Of the seven women with *T. vaginalis* detected, four were of black or mixed ethnicity (prevalence 2.7% (0.9% to 7.7%) in this group) and five reported recent partners of black or mixed ethnicity. Six of the women reported symptoms, and five reported sexual health clinic attendance in the past 5 years (prevalence in those reporting clinic attendance: 1.0% (0.4% to 2.3%)). The prevalence of a self-reported history of *T. vaginalis* (past 5 years) was 0.1% (0.0% to 0.2%) in women and 0.0% (0.0% to 0.2%) in men aged 16–44 years.

**Conclusions:**

Our British population prevalence estimates indicate that *T. vaginalis* is a rare infection. These data support policies that restrict asymptomatic screening for *T. vaginalis* and suggest deployment of molecular tests should be focused within clinical settings and guided by symptoms and local demography.

## Introduction

*Trichomonas vaginalis* is a sexually transmitted protozoan, which adheres to mucous membranes of the vagina and male urethra to cause trichomoniasis. Globally, *T. vaginalis* is the single most prevalent non-viral STI. Indeed, the WHO estimated that more than 270 million cases occurred in 2008, greater than the combined total for chlamydia and gonorrhoea.^1^ However, *T. vaginalis* diagnosis is relatively rare in the UK, with around 6000 cases reported each year, compared with over 200 000 chlamydia cases.^2^ ^3^

National STI surveillance data in England show that over 90% of diagnosed *T. vaginalis* cases occur in women,^2^ ^3^ a gender disparity attributed to more rapid spontaneous clearance of infection in men. Between 2009 and 2011, just under half of *T. vaginalis* diagnoses were in women of white ethnicity (in whom the rate was 10.7/100 000 population), but the rate of *T. vaginalis* diagnoses was much higher in black Caribbean women (329.6/100 000) and women of other black ethnicities (498.4/100 000), and much lower in men (33.9/100 000 in black Caribbean men and 0.6/100 000 in white men).^2^ However, diagnosis data are limited for a number of reasons, including that asymptomatic individuals may not seek healthcare, so the extent to which there might be a pool of undiagnosed infection in the wider population is unknown.

The BASHH 2014 guidelines recommend testing women according to clinical signs and symptoms, and men with persistent urethritis and/or who are contacts of individuals diagnosed with *T. vaginalis*.^4^ Widespread screening for *T. vaginalis* was previously limited because of a reliance on diagnostic assays, such as culture or wet mounts, which lack sensitivity and are time consuming. However, molecular assays with enhanced sensitivity are now readily available, either commercially or in-house, raising important questions about deployment of such tests for diagnostic and screening purposes.

The third National Survey of Sexual Attitudes and Lifestyles (Natsal-3) is a probability sample survey, which included urine testing and reported history of diagnoses to estimate the prevalence for a range of STIs.^5^ Natsal-3 used molecular assays for *T. vaginalis* to enable description of the age-specific and gender-specific prevalence of *T. vaginalis* within the sexually active British general population to inform clinical practice and public health policy.

## Methods

### Participants and survey procedures

Natsal-3 was a stratified probability sample survey of 15 162 men and women aged 16–74 years in Britain who were interviewed during 2010–2012.^6^ ^7^ The estimated overall response rate was 57.7%, and the cooperation rate was 65.8% (of all eligible addresses contacted). Participants were interviewed in their own homes using computer-assisted personal interview and computer-assisted self-interview (CASI). The CASI included questions about participants' sexual behaviour, their history of being diagnosed with STIs by a healthcare professional and their experience of genital symptoms associated with STIs in the month before interview. After the interview, we invited a sample of participants aged 16–44 years to provide urine for STI testing. Full methodological details have been described elsewhere.^5–7^

### Laboratory methods

In 2013, we undertook anonymous testing for *T. vaginalis*, without return of results, on DNA extracts taken from urine samples where there was sufficient sample (4482 out of 4550 participants) and where participants had provided consent for storage (4386 out of 4482 participants). All DNA extracts were screened using a modified primary RT-PCR that detects a 92-bp repeat-region fragment of *T. vaginalis* DNA,^8^ ^9^ and samples that generated a positive or equivocal screening result were retested with a secondary RT-PCR, which targets a conserved portion of the *T. vaginalis* β-tubulin gene.^9^ ^10^ A confirmed positive result was deemed to be one where the first RT-PCR was positive or equivocal and the second RT-PCR was positive.

### Statistical analysis

Prevalence estimates with 95% CIs in women and men are reported by age group for *T. vaginalis* detected in urine, and self-reported *T. vaginalis* diagnoses over the lifetime and in the past 5 years. Survey analyses were done in Stata V.13 accounting for sample stratification, clustering and weighting. Analyses were additionally weighted for unequal urine selection probabilities and differential urine sample response.^5–7^

## Results

Urinary *T. vaginalis* test results were available from 4386 sexually active participants (2559 women; 1827 men; 54.5% of all eligible participants) aged 16–44 years. The primary screening test detected *T. vaginalis* DNA in seven samples, all of which were confirmed with the secondary test. All seven samples were from women (there were no positive tests in men), giving a weighted prevalence estimate of 0.3% (95% CI 0.1% to 0.5%) in women aged 16–44 years (table 1). The prevalence of *T. vaginalis* in women decreased with age group, with the highest prevalence (0.6% (0.2% to 1.7%)) in those aged 16–24 years. Two women with *T. vaginalis* were coinfected with chlamydia, and two had at least one human papilloma virus-type detected; none of the women had *Neisseria gonorrhoeae*, HIV or *Mycoplasma genitalium* detected.^5^ ^w1 w2^

**Table 1:** Prevalence of *T. vaginalis* in urine in participants aged 16–44 years, by age group and gender

	Women		Men		Denominator women		Denominator men	
	Per cent	CI	Per cent	CI	Unwt	Wt	Unwt	Wt
16–24	0.6	(0.2% to 1.7%)	0		958	576	818	604
25–34	0.3	(0.1% to 1.0%)	0		1097	774	668	778
35–44	0.0		0		504	838	341	809
Total	0.3	(0.1% to 0.5%)	0		2559	2188	1827	2191

Unwt, unweighted; wt, weighted.

Although small numbers prevent detailed characterisation of cases, we observed that four of the seven women were of black or mixed ethnic origin, giving a weighted prevalence of 2.7% (0.9% to 7.7%) in this broad ethnicity group. All seven women reported at least one opposite sex partner in the past 5 years. The four women of black or mixed ethnicity and one white woman reported partners who were of black, black British or mixed ethnicity. Five of the women reported attending a sexual health clinic in the past 5 years (for three, this was in the past year), giving an estimated weighted prevalence of 1.0% (0.4% to 2.3%) among clinic attendees (past 5 years). Six of the women reported symptoms (abnormal or odorous vaginal discharge, or lower abdominal, or pelvic pain) in the month before interview. None reported a previous diagnosis of *T. vaginalis*.

Of 13 658 Natsal participants aged 16–74 years who reported at least one lifetime partner, 0.5% (0.3% to 0.7%) of women and 0.0% (0.0% to 0.2%) of men reported a *T. vaginalis* diagnosis in their lifetime (corresponding to 36 women and 3 men) (table 2). Among women, reported lifetime diagnosis of *T. vaginalis* was more common in those aged 45–74 years (0.8% (0.5% to 1.2%) compared with those aged 16–44 years (0.2% (0.1% to 0.4%); p=0.0018). However, diagnosis in the past 5 years was only reported by younger people (six women and two men), with an estimated prevalence in those aged 16–44 years of 0.1% (0.0% to 0.2%) in women and 0.0% (0.0% to 0.2%) in men.

**Table 2:** Self-reported history of *T. vaginalis* in participants aged 16–74 years, by age group, gender and timeframe

	Women		Men		Denominator women		Denominator men	
	Per cent	CI	Per cent	CI	Unwt	Wt	Unwt	Wt
Lifetime diagnoses								
16–24	0.1	(0.0% to 0.4%)	0.1	(0.0% to 0.8%)	1726	962	1363	990
25–34	0.3	(0.1% to 0.6%)	0.1	(0.0% to 0.4%)	2362	1304	1431	1281
35–44	0.3	(0.1% to 0.9%)	0.1	(0.0% to 0.8%)	1169	1397	775	1367
45–54	0.8	(0.4% to 1.5%)	0.0		1058	1365	745	1336
55–64	0.8	(0.4% to 1.7%)	0.0		969	1169	692	1085
65–74	0.7	(0.3% to 1.7%)	0.0		787	843	581	758
Total aged 16–44	0.2	(0.1% to 0.4%)	0.1	(0.0% to 0.3%)	5257	3662	3569	3639
Total aged 45–74	0.8	(0.5% to 1.2%)	0.0		2814	3377	2018	3180
Total	0.5	(0.3% to 0.7%)	0.0	(0.0% to 0.2%)	8071	7040	5587	6818
Past 5 years								
16–24	0.1	(0.0% to 0.4%)	0.1	(0.0% to 0.8%)	1726	962	1363	990
25–34	0.1	(0.0% to 0.4%)	0.1	(0.0% to 0.4%)	2362	1304	1431	1281
35–44	0.0	0.0%	0.0	0.0%	1169	1397	775	1367
45–54	0.0	0.0%	0.0	0.0%	1058	1365	745	1336
55–64	0.0	0.0%	0.0	0.0%	969	1169	692	1085
65–74	0.0	0.0%	0.0	0.0%	787	843	581	758
Total aged 16–44	0.1	(0.0% to 0.2%)	0.0	(0.0% to 0.2%)	5257	3662	3569	3639
Total aged 45–74	0.0		0.0		2814	3377	2018	3180
Total	0.0	(0.0% to 0.1%)	0.0	(0.0% to 0.1%)	8071	7040	5587	6818

Unwt, unweighted; wt, weighted.

## Discussion

This study provides the first population-based prevalence estimates for *T. vaginalis* in the British general population and indicates that *T. vaginalis* is an uncommon infection. A molecular assay with confirmatory testing was used, and we detected *T. vaginalis* in only a small number of women in a large sample, which was broadly representative of the sexually active general population. All cases except two were in women of Black or mixed ethnicity, or reported recent partners of Black or mixed ethnicity, and most had symptoms consistent with infection. Most had recently attended a sexual health clinic. These data are supported by our finding that the prevalence of reported *T. vaginalis* diagnosis in the past 5 years in those aged 16–44 year was also very low.

In this cross-sectional study, the use of urine, which is a suboptimal specimen for the detection of *T. vaginalis*, particularly in men, might have led to underestimation of prevalence.^w3 w4^ DNA extracts were stored for up to 3 years before testing, introducing a small risk of degradation, which might have reduced assay sensitivity. While the size and representative nature of the sample are important strengths of this study, Natsal-3 did not include an ethnic boost, and the total number of non-white participants was relatively small. This meant that subanalyses were not possible, and underrepresentation of these groups might also have led to underestimation of prevalence.

Worldwide data sets providing robust estimates of *T. vaginalis* prevalence in the general population are limited to the USA. The 2001–2002 Add Health study found the prevalence in women aged 18–26 years was 2.8% (2.2% to 3.6%).^w5^ The 2001–2004 NHANES study reported that the prevalence in women aged 14–49 years was 3.1% (2.2% to 4.3%), driven by very high prevalence in women of non-Hispanic black ethnicity (13.3% (10.0–17.7).^w6^ A probability sample of young adults undertaken in Baltimore in 2006–2009 reported that prevalence was 4.1% (2.3% to 7.0%) in non-black women and 16.6% (13.0% to 19.8%) in women of black ethnicity.^w7^ The use of different samples and assays in these studies complicates comparison, but it is striking that the US data consistently show much higher prevalence estimates than we found in Britain. Differences in the demographic composition of the USA and British populations might partially account for these findings, but we note that prevalence estimates in black women in the Natsal sample are also lower, which might be explained by differences in access to healthcare. Overall higher rates of STIs found in black populations might also be due to differences in sexual behaviour (eg, overlapping partnerships and assortative mixing), and associated with underlying social factors such as deprivation.^w8^

These data are timely because molecular assays to detect *T. vaginalis* are now commercially available, increasing the feasibility of widespread screening, including outside of specialist sexual health services. However, given the very low prevalence detected, unselected screening of asymptomatic individuals is unlikely to represent a cost-effective use of resources. Instead, these data support policies that focus the deployment of molecular tests within clinical settings where pilot studies and/or demography provide evidence of higher prevalence in the local community. It is now the role of professional clinical and public health organisations to take these findings, together with other published evidence, to make recommendations about *T. vaginalis* screening and diagnostic testing.

### Ethical approval

We obtained ethics approval from Oxfordshire Research Ethics Committee A (reference 09/H0604/27). Participants gave written informed consent to anonymised testing, without the return of results, the ethical rationale for which has been previously described.^w9^ A substantial amendment was subsequently approved by Oxfordshire Research Ethics Committee to test for *T. vaginalis* where participants had provided consent to any remaining urine being stored for studies investigating diseases in the population.[Supplementary-material SM1]

10.1136/sextrans-2016-052660.supp1 Supplementary references
