# Supplementary material for: Trichomonas vaginalis infection is uncommon in the British general population: implications for clinical testing and public health screening
Source: Sex Transm Infect. 2016 Sep 29;94(3):226–9. doi: 10.1136/sextrans-2016-052660 (PMC5969328; doi:10.1136/sextrans-2016-052660)
Supplement: Supplementary references [file sextrans-2016-052660supp001.pdf]

Web appendix – references continued

w1 Sonnenberg P, Ison CA, Clifton S, *et al.* Epidemiology of Mycoplasma genitalium in British men and women aged 16-44 years: evidence from the third National Survey of Sexual Attitudes and Lifestyles (Natsal-3). *Int J Epidemiol* Published Online First: 3 November 2015. doi:10.1093/ije/dyv194

w2 Field N, Clifton S, Alexander S, *et al.* Confirmatory assays are essential when using molecular testing for Neisseria gonorrhoeae in low-prevalence settings: insights from the third National Survey of Sexual Attitudes and Lifestyles (Natsal-3). *Sex Transm Infect* 2015;**91**:338–41. doi:10.1136/sextrans-2014-051850

w3 Lawing LF, Hedges SR, Schwebke JR. Detection of Trichomonosis in Vaginal and Urine Specimens from Women by Culture and PCR. *J Clin Microbiol* 2000;**38**:3585–8.

w4 Hobbs MM, Lapple DM, Lawing LF, *et al.* Methods for detection of Trichomonas vaginalis in the male partners of infected women: implications for control of trichomoniasis. *J Clin Microbiol* 2006;**44**:3994–9. doi:10.1128/JCM.00952-06

w5 Miller WC, Swygard H, Hobbs MM, *et al.* The prevalence of trichomoniasis in young adults in the United States. *Sex Transm Dis* 2005;**32**:593–8.

w6 Allsworth JE, Ratner JA, Peipert JF. Trichomoniasis and other sexually transmitted infections: results from the 2001-2004 National Health and Nutrition Examination Surveys. *Sex Transm Dis* 2009;**36**:738–44. doi:10.1097/OLQ.0b013e3181b38a4b

w7 Rogers SM, Turner CF, Hobbs M, *et al.* Epidemiology of undiagnosed trichomoniasis in a probability sample of urban young adults. *PloS One* 2014;**9**:e90548. doi:10.1371/journal.pone.0090548

w8 Hughes G, Field N. The epidemiology of sexually transmitted infections in the UK: impact of behavior, services and interventions. *Future Microbiol* 2015;**10**:35–51. doi:10.2217/fmb.14.110

w9 Field N, Tanton C, Mercer CH, *et al.* Testing for sexually transmitted infections in a population-based sexual health survey: development of an acceptable ethical approach. *J Med Ethics* 2012;**38**:380–2. doi:10.1136/medethics-2011-100068
